# Evaluation of natural head position over five minutes: A comparison between an instantaneous and a five‐minute analysis with an inertial measurement unit

**DOI:** 10.1111/joor.13297

**Published:** 2022-01-12

**Authors:** Mustafa Al‐Yassary, Kelly Billiaert, Gregory S. Antonarakis, Stavros Kiliaridis

**Affiliations:** ^1^ Division of Orthodontics University Clinics of Dental Medicine University of Geneva Geneva Switzerland

**Keywords:** Clinicians, head, position, sitting, wearable electronic devices

## Abstract

**Background:**

Head posture is a balance of several positions and therefore shows inherent variation. Most methods available to quantify this are however instantaneous, not providing information about its variation over time. A dynamic recording of head posture would thus be beneficial.

**Objectives:**

The purpose of this study was to evaluate the variation in natural head position (NHP) over 5 min using an inertial measurement unit (IMU).

**Methods:**

Fifteen healthy young volunteers were asked to sit on a chair and keep their head in the self‐balanced position for 5 min. A mirror was then revealed in front of them, and they were asked to look at their eyes for 20 s. This procedure was undertaken on two separate occasions with a one‐week interval. This was compared to an instantaneous measurement of head position at a specific time point corresponding to the 15th second of the recording.

**Results:**

During the 5 min of recording, the participants tended to elevate their head progressively by a mean of 1.5°, which is then corrected by looking at oneself in the mirror. Most participants tended to rotate their head to the left and continued that progressive rotation despite looking in the mirror. The roll axis had no systematic changes observed between the self‐balanced position and the mirror‐guided position and was the most reproducible axis. Moderate to good correlations were found comparing both sessions for each axis.

**Conclusion:**

The comparison between the five‐minute analysis and the instantaneous measurement showed a systematic difference on the pitch axis but no differences for the yaw and roll. These results suggest that the variation in the NHP during a period of 5 min is generally specific to each participant with a head elevation and rotation to the left in most cases.

## Graphical abstract

Natural Head Posture (NHP) is a balance of several positions and therefore has inherent variation. However, most measurement methods available are instantaneous, not providing information about variation over time. A continuous (dynamic) recording could allow a better evaluation of head posture. The objective of this study was to evaluate the variation in NHP over 5 min using an Inertial Measurement Unit. We found that NHP variations are generally specific to each participant. And Systematic differences on the pitch axis were observed when comparing continuous and instantaneous measurements. We also found that the evolution of the head posture was generally similar among the participants.

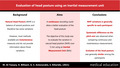

## 1 INTRODUCTION

Most methods available to measure the natural head position (NHP)^1^ use an instantaneous measurement corresponding to one position at a specific point in time. However, the NHP is known to be a balance of several positions^2^; therefore, it is not a fixed position, and inherent variation is present for each person. The characteristics and implications of this variation are not fully understood. The head position is related to different alterations in fields such as orthodontics,^3^ orthopaedics,^4^ respiratory physiology,^5^, ^6^ and ophthalmology.^7^

To measure the NHP, there are two main standard positions, namely, the self‐balanced position and the self‐guided position. The self‐balanced position is adopted when a person looks at an imaginary point in front of him/her, from an infinite distance at the eye level.^8^ The self‐guided position is when a person looks at themselves in a mirror.^8^ Different studies indicate that the mirror‐guided head position is the most stable and reproducible^9^, ^10^; however, it is rarely adopted in everyday life. It is thus preferable, in order to more accurately simulate the head position in a natural environment, to analyse the self‐balanced head position that is more frequently adopted. In growing individuals, there are hypotheses that the head position may influence the craniofacial growth pattern.^8^, ^11^ In such cases, it is possible that the evaluation of the NHP during a longer period could be of greater interest rather than solely looking at the head position at a specific point in time.

**FIGURE 1:**
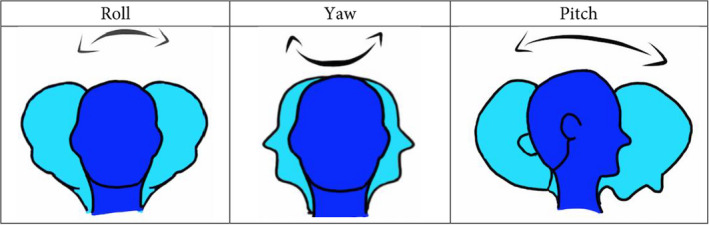
Representation of head movements: showing the representation of the head movements on the three axes, namely, the pitch axis, roll axis, and yaw axis

Evaluation of the NHP is performed on three axes namely pitch, roll, and yaw. The pitch is the angle between the horizontal plane and the Frankfort plane. The roll is the angle between the horizontal plane and the bipupillary plane. Finally, the yaw is the angle between the acromial process and the median sagittal plane. (Figure 1).

The aims of this study were to

1. Compare the instantaneous analysis to a five‐minute analysis of the head position.
2. Analyse the evolution of the NHP in a self‐balanced position for 5 min and compare it to the mirror‐guided head position for the three axes.
3. Evaluate the reproducibility of the self‐balanced head posture and the mirror‐guided head position for the three axes in a group of healthy young adults.

## 2 METHODS

### 2.1 Ethics statement

The present study was approved by the Swiss Association of Research Ethics Committee. The experimental procedures were conducted in conformity with the Declaration of Helsinki. Informed consent for participation in the study and publication in an open‐access format was obtained from the participants, with regard to their recordings and personal information. The procedures of the study were fully explained to the participants, and they provided their informed written consent prior to being enrolled in the study.

### 2.2 Subjects and methods

Fifteen healthy young adult volunteers (eight women and seven men) at the University Clinics of Dental Medicine in Geneva, Switzerland, aged from 20–30 years (mean age of 28.4 years) were recruited for this study.

Wearable sensors or inertial measurement units (IMU)s were used to measure head position. These are electronic devices allowing movements to be tracked in three dimensions (3D).^12^ This system was chosen because it is easy to use, precise,^13^ and allows tracking of head position in a given period. This system is composed of a gyroscope, an accelerometer, and a magnetic angular rate and gravity. The hardware consists generally of two parts, a detector fixed on the element of interest and a receiver. The information is stored to allow one to access the recordings of the patients and to follow them.^14^ An IMU allows the head position to be recorded dynamically for a prolonged time period, thus it is possible to analyse the full variation of the NHP during a 5‐minute recording.

The participants were asked to sit on a chair in the 90–90–90 position (corresponding to a 90° angle on the hips, the knees, and the feet)^15^ facing a white wall which was one meter in front of them. They were asked to look in front of them and to keep a straight head posture with their own feeling of natural head balance (self‐balanced position). This position was recorded for 5 min while listening to a short story played behind them with stereo‐speakers carefully centred on the volunteers. Following this, a mirror was placed on the wall in front of the participants, and they were asked to look at their eyes for 20 s (self‐guided position). This procedure was repeated twice with a one‐week interval, and during the second recording, a different short story was played in order to allow for a similar experimental setup without the participants having to listen to the same story twice.

The IMU system used was the MetaMotionR (Mbientlab lnc.). This system is composed of two sensors. Before measuring the head position, this system needs to be calibrated to assess the neutral position (corresponding to 0° on each axis). This was done by placing the first sensor on the ground in front of the participant and the second one behind them. Both have to be parallel, on the same plane, and perpendicular to the ground axis. After calibration, the first sensor was placed on the forehead of the participant.

In order to minimize the error due to the positioning of the sensor, one front face photograph was taken to make sure that the sensor was parallel to the bipupillary line. A second profile photograph was taken to eliminate the slope of the forehead from the IMU recordings. The postprocessing calibration was performed with Pixelstick (version 2.16.2, Plum Amazing software LLC). This calibration method was the same as that used by Billiaert et al.^16^ that compared the IMU system to lateral photographs and showed excellent accuracy.

### 2.3 Statistical analysis

**FIGURE 2:**
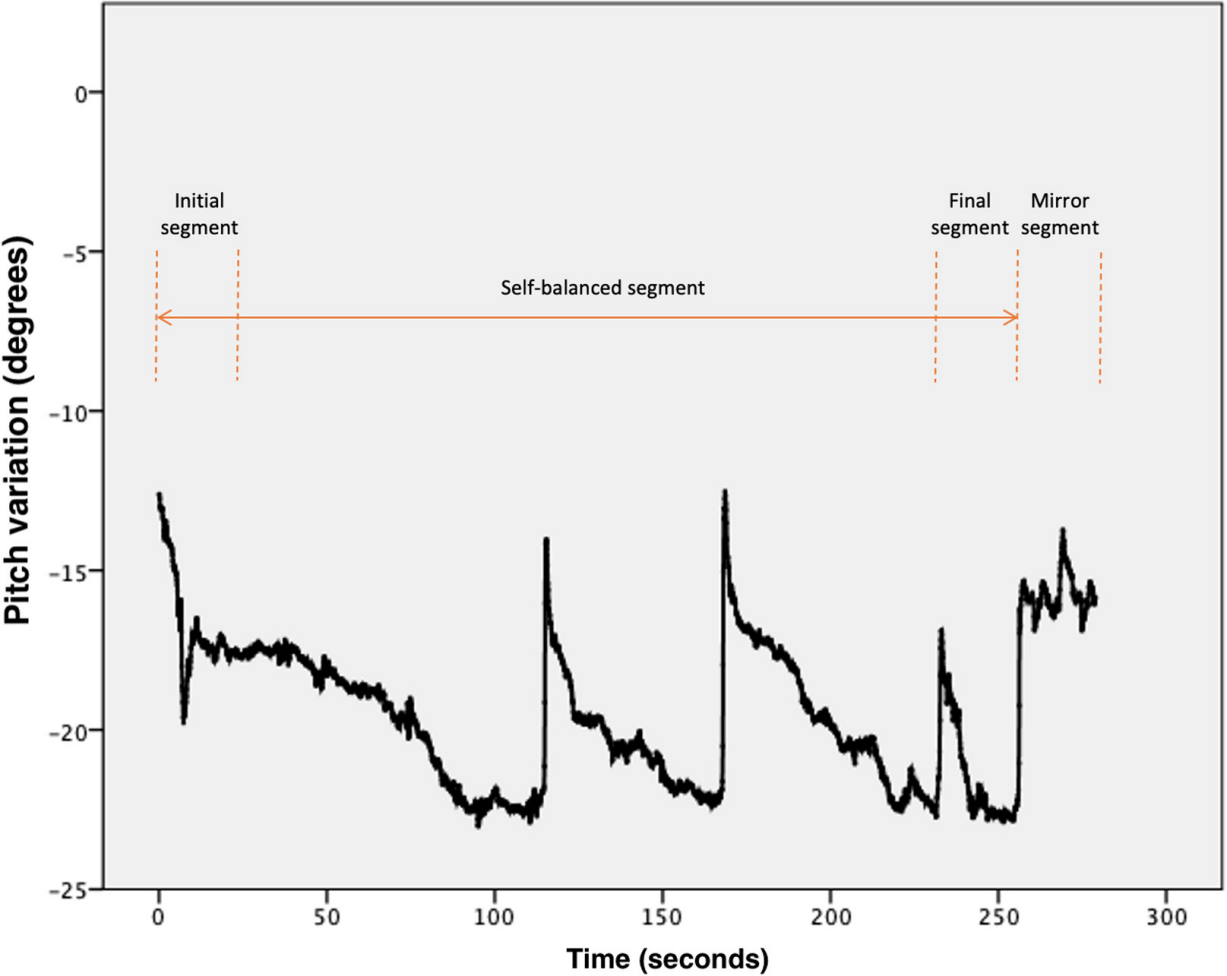
Representation of the variation of the natural head position on the pitch axis for five minutes showing the variation of head position for the pitch axis. The orange arrow represents the self‐balanced segment of the recording with the initial segment at the beginning and the final segment at the end. The mirror segment (mirror‐guided position) is represented following the self‐balanced segment

All statistical analyses were performed using SPSS (version 24.0, SPSS Inc.). The data were separated into four different segments. The initial segment (I) represents the mean of the first 20 s, the finishing segment (F) the mean of the last 20 s before the mirror‐guided position. The self‐guided position (S) is the mean of the full segment from initial to finish. Finally, the mirror‐guided position segment (M) represents the mean of the 20 s when the mirror was revealed in front of the participants (Figure 2). The means of 20 s were taken for the I, F, and M segments in order to have a more reliable representation of the head position. In total, for each participant, 24 means were calculated (two sessions, four segments for each session, and three axes for each segment). The extreme movements (outliers) that were not related to the NHP (for example if a participant sneezes, coughs, or moves their arms or feet) were eliminated.

For the instantaneous measurement, recorded data were taken at the 15th second with the IMU system. As there are no clear instructions in previous studies regarding the time at which the photograph for the head position measurement was taken, the 15th second was chosen because it represents approximatively the time required to take a photograph.

The differences between the instantaneous measurement and the five‐minute recordings were analysed. The instantaneous measurement was taken and compared to the mean of the recordings for the self‐balanced head position (S). The difference between I–F, F–M, and I–M was analysed to better understand the evolution and tendency of the head position. The self‐guided head position (S) was also compared to the mirror‐guided head position (M), and the two sessions were compared to assess whether the variation within the five‐minute recording period was random or if it followed a specific and reproducible pattern.

For each set of measurements, the difference between the means was calculated, with *p* values inferior to .05 considered statistically significant. Pearson's correlations and intraclass correlation (ICC)*^17^ with 95% confidence intervals (95% CI) (based on a mean‐rating (k = 2), absolute‐agreement, 2‐way mixed‐effects model) were also calculated. ICC values less than 0.5 are indicative of poor reliability, values between 0.5 and 0.75 indicate moderate reliability, values between 0.75 and 0.9 indicate good reliability, and values greater than 0.90 indicate excellent reliability.^18^ The standard error measurement (SEM) was calculated for each ICC (using the formula $\text{SEM}=Standard\;Deviation\times \sqrt{1‐ICC}$).

## 3 RESULTS

The data show a greater interindividual variation in the pitch axis with a negative value corresponding to an elevated Frankfort plane than the horizontal plane. The roll axis had the least amount of interindividual variation and a slight deviation to the right compared to the median plane. The yaw axis had comparable interindividual variation to the roll axis and had a slight deviation to the left compared to the plane that passes by the acromial process (shoulders) (Table 1).

**TABLE 1:** Summary of the first session of the study population head position measured with an inertial measurement unit

*N *= 15	Pitch	Roll	Yaw
Mean ± SD	−16.8° ±4.6	−1.5° ±3.2	1.5° ±3.0
95% CI	−19.1 – −14.5	−3.1 – 0.1	0 – 3.0

Note

The recordings correspond to an instantaneous analysis of the head position in a sitting and self‐balanced position. Shown are the number participant (*n*), the mean, standard deviation (SD), and 95% confidence interval (95% CI) of the recordings for each axis.

### 3.1 Comparison of the one‐shot procedure to the five‐minute analysis

When comparing the instantaneous analysis to the mean of the five‐minute analysis, a systematic difference was found for the pitch axis (*p* = .04), whereby the mean pitch from the five‐minute procedure was elevated by 0.9 degrees compared to the instantaneous procedure. For the roll and yaw axes, no systematic differences (*p* > .05) were found. However, a good correlation and good to excellent reliability was found for all the three axes (Table 2).

**TABLE 2:** Comparison between an instantaneous and a five‐minute recording of head position using an inertial measurement unit

	Pitch	Roll	Yaw
Mean ± SD (*p*‐Value)	0.9° ±2.2 (0.04)	0.1° ±1.2 (0.85)	−0.6° ±2.7 (0.2)
r (*p*‐Value)	0.93 (0.00)	0.92 (0.00)	0.79 (0.00)
ICC (95% CI)	0.95 (0.89–0.98)	0.96 (0.91–0.98)	0.86 (0.71–0.93)
SEM	0.48	0.25	1.00

Note

Shown are the mean and standard deviation (SD) of the difference (T^1^–T^2^) between the instantaneous (corresponding to T^1^) and the five‐minute recording (corresponding to T^2^), Pearson's correlation (r), the intraclass correlation coefficient (ICC)^17^ with 95% confidence interval (95% CI), and their standard error measurement (SEM) for each axis.

### 3.2 Analysis of the evolution of the NHP in a self‐balanced position for five minutes compared with the mirror‐guided head position for the three axes

During the self‐balanced position, the participants tend to elevate their head by 1.5° ±3.8° (*p* = .03), which was then corrected by the mirror‐guided position (−1.3°± 4.2°; *p* = .013). The roll axis has the least amount variation with the standard deviation ranging from 0.9° to 2.3°. On the yaw axis, the participants tend to progressively increase the deviation, generally to the left by 2.3° ±4.7° (*p* = .13), and the mirror‐guided position does not seem to correct this.

Overall, great correlation was found between the different segments for the pitch and roll axes ranging from 0.72 to 0.87. However, for the yaw axis, the correlations were lower, ranging from 0.49 to 0.93 due to higher deviations. For the pitch and roll axes, good‐to‐excellent reliability was found between the segments (ranging from 0.83 to 0.93), and for the yaw axis moderate to excellent reliability was found (ranging from 0.55 to 0.96).

### 3.3 Evaluation of the reproducibility of the self‐balanced and the mirror‐guided head position

**FIGURE 3:**
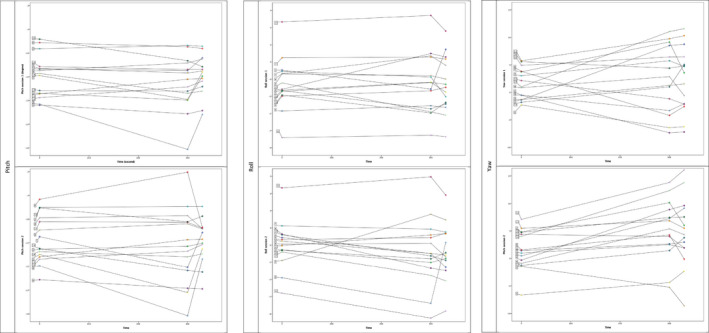
Spaghetti plots showing the evolution of the head position over a five‐minute recording period for the three axes: The X‐axis represents the time of recording in seconds. The Y‐axis represents the intensity of the changes of the head position. For each participant, three means are represented by points on this plot (the initial segment corresponding to the mean of the first 20 seconds of the recording, the final segment corresponding to the mean of the last 20 seconds before the mirror‐guided position, and the mirror segment corresponding to the mean of the mirror‐guided segment)

No systematic differences were found between the two sessions for each segment (*p* > .05). When comparing the same segments for the two different sessions (Figure 3), moderate to good correlations were found depending on the axis. The pitch axis had the strongest correlations followed by the roll and the least reproducible axis was the yaw. Good to excellent reliability was found for the self‐balanced head position (ranging from 0.78 to 0.96) and poor‐to‐excellent reliability for the mirror‐guided head position (ranging from 0.49 to 0.92) depending on the axis (Table 3).

**TABLE 3:** Comparison between the two sessions of the self‐balanced head position and the mirror‐guided head position

Segments		Pitch	Roll	Yaw
Self‐ balanced	Mean ± SD (*p*‐Value)	−0.6° ±2.2 (0.35)	−0.3° ±1.6 (0.53)	1.6° ±3.5 (0.11)
	r (*p*‐Value)	0.90 (0.00)	0.72 (0.00)	0.69 (0.01)
	ICC (CI 95%)	0.96 (0.89–0.99)	0.94 (0.82–0.98)	0.78 (0.38–0.93)
	SEM	0.43	1.39	1.65
Mirror	Mean ± SD (*p*‐Value)	0.0° ±2.4 (0.96)	0.1° ±2.0 (0.89)	−2.4° ±6.9 (0.21)
	r (*p*‐Value)	0.85 (0.00)	0.58 (0.24)	0.34 (0.22)
	ICC (CI 95%)	0.92 (0.76–0.97)	0.75 (0.21–0.92)	0.49 (0.41–0.82)
	SEM	0.68	1.22	4.93

Note

The self‐balanced segment represents the mean of the five‐minute recording of the head position in the self‐balanced head posture, and the mirror segment represents the mean of a 20‐second recording for the mirror‐guided head posture. For each segment and axis, the first to the second session was compared. Shown are the mean and standard deviation (SD) of the difference between the two sessions, Pearson's correlation coefficient (r), the intraclass correlation coefficient (ICC)^17^ with 95% confidence interval (95% CI), and their standard error measurement (SEM) for each axis.

## 4 DISCUSSION

The present study showed an excellent correlation between the instantaneous procedure and the mean of the five‐minute recordings for the pitch and roll axes and good correlation for the yaw axis. The five‐minute analysis showed a systematic elevation of the head compared to the instantaneous procedure, but no systematic changes for the roll and yaw axes were observed.

The data recorded at the 15th second with the IMU system were used as a representative instantaneous procedure. This time was chosen because it represents approximately the time required to take a photograph. Moreover, unlike photography, the IMU system allows one to obtain data for all three axes. The study of Billiaert et al.^16^ as well as Al‐Yassary et al.^19^ demonstrated excellent accuracy of the IMU system for measuring head position.

When the different segments were compared, it was found that the participants tended to elevate their heads and turn progressively to the left. However, a general trend for the roll axis was not found. When the mirror was displayed, it was observed that the participants corrected their elevation on the pitch axis, without any effects on the other two axes (roll and yaw). The participants that tended to rotate their head kept their rotation and continued in the same direction.

The variation of head position seems to be influenced by different factors. The rotation of the head may be related to the dominant eye, which is often the right eye. This observation is described by Pradham et al. who found a strong relationship between eye dominance and direction of head rotations.^20^ It was also observed that a progressive elevation in the self‐balanced head position occurs over time to a maximum of 1.5° after 5 min, likely caused by muscle fatigue. This elevation of the head in the self‐balanced head position compared to the mirror‐guided head position has also been observed by Jakobsone et al.^9^

When comparing the two sessions, a stronger correlation was found for the self‐balanced head position than the mirror‐guided head position on each axis. The correlations of the mirror‐guided head position found in this study were similar to the ones found by Billiaert et al.^16^ who analysed the reproducibility of the head position in a sitting and standing position. When comparing the two sessions, the standard deviation (SD) found for the mirror‐guided head posture on the pitch axis (SD = 2.4°) in this study is similar to the one found by Peng and Cooke (SD = 4.3°) for a fifteen‐year reproducibility of natural head posture.^10^

For the mirror‐guided head position, a good correlation was observed on the pitch axis since the mirror affects it. However, the mirror‐guided position does not seem to influence the roll and yaw axes, which is why the correlation was much lower for these axes. For the self‐balanced head position, good correlation was found even for the roll and the yaw axes. This indicates a similar pattern between the two sessions, meaning that the variation in head position may be specific to each participant.

The limitations in the present study include the potential lack of generalizability as all the included participants were healthy young adults and, thus, represent only a small part of the population. However, the use of this system can be interesting for every population including children, the elderly, and patients with a handicap or other pathological conditions since it is easy to use and allows a recording of the variations of head position over a certain period of time.

The reproducibility found in this study is based on a one‐week interval, and on two sessions. A longer period between the recordings and multiple sessions would be necessary to evaluate the long‐term reproducibility. Finally, different elements influence the head position, such as fatigue and ocular and respiratory factors, and thus the recordings of each participant should be evaluated when these factors are the same for the two sessions.

## 5 CONCLUSION

Good‐to‐excellent correlations were found between the instantaneous analysis of head position and the five‐minute head position recordings. However, on the pitch axis, the head position was systematically more elevated in the five‐minute analysis than the instantaneous procedure, whereas no difference was observed for the roll and yaw axes.

Most participants have the same pattern of evolution of their head position over a five‐minute recording. An elevation of the head and a rotation to the left were observed as general patterns. Only the elevation of the head, however, is corrected by the mirror‐guided position.

The pattern of evolution of the head position and its variation are generally specific to each participant with moderate‐to‐good correlation between the two sessions. Overall, the means of the five‐minute recordings were more reproducible than those of the mirror‐guided head position. The roll axis had the least amount of variation, followed by the pitch and finally the yaw axes, respectively.

## CONFLICT OF INTEREST

The authors declare no competing interests.

## AUTHOR CONTRIBUTIONS

K.B. and M.A., contributed to the data collection and data analysis, G.S.A. and S.K. contributed to the study design and drafting of the manuscript.

### PEER REVIEW

The peer review history for this article is available at https://publons.com/publon/10.1111/joor.13297.

## Data Availability

All data included in this study are available upon reasonable request from the corresponding author.

## References

[1] Broca M . Sur les projections de la tète, et sur un nouveau procède de cephalometrié. Bull Soc Anthropol Paris. 1862;3:514‐544.

[2] Lundstrom A , Forsberg CM , Westergren H , Lundstrom F . A comparison between estimated and registered natural head posture. Eur J Orthod. 1991;13:59‐64. doi:10.1093/ejo/13.1.59 2032569

[3] Solow B , Sonnesen L . Head posture and malocclusions. Eur J Orthod. 1998;20:685‐693. doi:10.1093/ejo/20.6.685 9926635

[4] Nucci P , Kushner BJ , Serafino M , Orzalesi N . A multi‐disciplinary study of the ocular, orthopedic, and neurologic causes of abnormal head postures in children. Am J Ophthalmol. 2005;140:65‐68. doi:10.1016/j.ajo.2005.01.037 16038652

[5] Cuccia AM , Lotti M , Caradonna D . Oral breathing and head posture. Angle Orthod. 2008;78:77‐82. doi:10.2319/011507-18.1 18193952

[6] Huggare JA , Laine‐Alava MT . Nasorespiratory function and head posture. Am J Orthod Dentofacial Orthop. 1997;112:507‐511. doi:10.1016/s0889-5406(97)70078-7 9387837

[7] Nucci P , Curiel B , Lembo A , Serafino M . Anomalous head posture related to visual problems. Int Ophthalmol. 2015;35:241‐248. doi:10.1007/s10792-014-9943-7 24719022

[8] Solow B , Tallgren A . Head posture and craniofacial morphology. Am J Phys Anthropol. 1976;44:417‐435. doi:10.1002/ajpa.1330440306 937521

[9] Jakobsone G , Vuollo V , Pirttiniemi P . Reproducibility of natural head position assessed with stereophotogrammetry. Orthod Craniofac Res. 2020;23:66‐71. doi:10.1111/ocr.12344 31514260

[10] Peng L , Cooke MS . Fifteen‐year reproducibility of natural head posture: a longitudinal study. Am J Orthod Dentofacial Orthop. 1999;116:82‐85. doi:10.1016/s0889-5406(99)70306-9 10393584

[11] Huggare JA , Cooke MS . Head posture and cervicovertebral anatomy as mandibular growth predictors. Eur J Orthod. 1994;16:175‐180. doi:10.1093/ejo/16.3.175 8062857

[12] Ghislieri M , Gastaldi L , Pastorelli S , Tadano S , Agostini V . Wearable inertial sensors to assess standing balance: a systematic review. Sensors (Basel). 2019;19(19):4075. doi:10.3390/s19194075 PMC6806601 31547181

[13] Beange KHE , Chan ADC , Beaudette SM , Graham RB . Concurrent validity of a wearable IMU for objective assessments of functional movement quality and control of the lumbar spine. J Biomech. 2019;97:109356. doi:10.1016/j.jbiomech.2019.109356 31668717

[14] Patel S , Park H , Bonato P , Chan L , Rodgers M . A review of wearable sensors and systems with application in rehabilitation. J Neuroeng Rehabil. 2012;9:21. doi:10.1186/1743-0003-9-21 22520559 PMC3354997

[15] Engström B . Ergonomic Seating: a True Challenge: Seating and Mobility for the Physically Challenged: Risks & Possibilities when Using Wheelchairs. Posturalis Books; 2002.

[16] Billiaert K , Al‐Yassary M , Antonarakis GS , Kiliaridis S . Measuring the difference in natural head position between the standing and sitting positions using an inertial measurement unit. J Oral Rehabil. 2021;48:1144‐1149. doi:10.1111/joor.13233 34293214 PMC9290966

[17] Cacucci L , Ricci B , Moretti M , Gasparini G , Pelo S , Grippaudo C . Surgical orthodontic treatment of a patient affected by type 1 myotonic dystrophy (Steinert syndrome). Case Rep Dent. 2017;2017:7957961. doi:10.1155/2017/7957961 28642828 PMC5469988

[18] Koo TK , Li MY . A guideline of selecting and reporting intraclass correlation coefficients for reliability research. J Chiropr Med. 2016;15:155‐163. doi:10.1016/j.jcm.2016.02.012 27330520 PMC4913118

[19] Al‐Yassary M , Billiaert K , Antonarakis GS , Kiliaridis S . Evaluation of head posture using an inertial measurement unit. Sci Rep. 2021;11:19911. doi:10.1038/s41598-021-99459-7 34620956 PMC8497508

[20] Pradham NS , White GE , Mehta N , Forgione A . Mandibular deviations in TMD and non‐TMD groups related to eye dominance and head posture. J Clin Pediatr Dent. 2001;25:147‐155. doi:10.17796/jcpd.25.2.j7171238p2413611 11314215

